# Co-delivery of anti-inflammatory and antioxidant agents via polymersomes for osteoarthritis therapy

**DOI:** 10.3389/fphar.2025.1635761

**Published:** 2025-07-09

**Authors:** Mengjie Rui, Li Wang, Ke Mi, Yinfeng Li, Naying Fang, Yingying Ge, Qiuqi Feng, Yaqi Luo, Chunlai Feng

**Affiliations:** ^1^ School of Pharmacy, Jiangsu University, Zhenjiang, Jiangsu, China; ^2^ NHC Key Laboratory of Diagnosis and Therapy of Gastrointestinal Tumor, Gansu Provincial Hospital, Lanzhou, Gansu, China

**Keywords:** osteoarthritis, cordycepin, phenyboronic acid, compound combination, co-loaded polymersomes

## Abstract

**Background:**

Osteoarthritis (OA) is a chronic degenerative joint disease primarily driven by inflammation and oxidative stress. This study aimed to develop a polymersome-based co-delivery system encapsulating hydrophilic cordycepin and hydrophobic phenylboronic acid (PBA) to enhance their solubility, stability, and therapeutic efficacy against OA.

**Methods:**

Formulation parameters were optimized using a Taguchi orthogonal design to achieve high encapsulation efficiency, sustained drug release, and effective reactive oxygen species (ROS) scavenging. *In vitro* anti-inflammatory effects were evaluated in LPS-activated RAW 264.7 macrophages by assessing TNF-α, IL-1β, and extracellular ROS levels. Therapeutic efficacy was further validated in a papain-induced OA rat model treated with co-loaded polymersomes via intraperitoneal injection for four weeks, with joint swelling and serum cytokines monitored.

**Results:**

The optimized co-loaded polymersomes exhibited an average size of 101.03 ± 0.42 nm and a polydispersity index (PDI) of 0.248 ± 0.014. They demonstrated a H_2_O_2_-responsive compound release and potent ROS-scavenging ability. *In vitro*, the co-loaded polymersomes significantly reduced inflammatory cytokines and ROS levels. In OA rat model, co-loaded polymersomes led to the greatest reduction in cartilage damage and promoted cartilage regeneration compared to other treatment groups.

**Conclusion:**

This co-delivery system offered a sustained release profile, enhanced joint targeting, and reduced adverse effects, resulting in superior therapeutic outcomes compared to free compounds alone or their combination. These findings highlighted its potential as a promising therapeutic approach for OA management.

## 1 Introduction

Osteoarthritis (OA) is one of the most common chronic degenerative joint diseases, primarily affecting the elderly population (Cao et al., 2024; Perruccio et al., 2024). OA causes pain, joint swelling, and cartilage damage, leading to restricted joint mobility and significantly impacting patients’ quality of life. The etiology of OA is multifactorial, involving inflammation, oxidative stress, aging, obesity, and genetics (Michael et al., 2010; Yao et al., 2023). Current treatment for early-stage OA provides temporary pain relief, with many patients eventually progressing to advanced stage that require joint replacement surgery (Katz et al., 2021; Maqbool et al., 2021; Kennedy et al., 2022).

Inflammation and oxidative stress are major drivers of the pathophysiology of OA. Pro-inflammatory cytokines, such as interleukin-1β (IL-1β), tumor necrosis factor-α (TNF-α), and interleukin-17 (IL-17), are elevated in OA joints, promoting cartilage degradation and chronic joint inflammation (Wojdasiewicz et al., 2014; De Roover et al., 2023; Gaddala et al., 2024). Among these, IL-1β plays an important role by stimulating chondrocytes and synovial cells to produce matrix metalloproteinases (MMPs) (Jenei-Lanzl et al., 2019; Mukherjee and Das, 2024) and reactive oxygen species (ROS) (Lepetsos and Papavassiliou, 2016; Ni et al., 2021), thereby exacerbating cartilage damage. However, treatments targeting IL-1β have not fully inhibited the progression of OA, revealing the complex inflammatory pathways of the OA. Conversely, anti-inflammatory cytokines such as interleukin-10 (IL-10) exhibit protective effects (van Meegeren et al., 2012), suggesting the potential of combined therapeutic strategies to enhance cartilage repair.

Excessive reactive oxygen species (ROS) production at OA sites significantly exacerbates the progression and severity of OA (Li et al., 2012; Lepetsos and Papavassiliou, 2016). The major source of ROS is the mitochondrial and NADPH oxidases (NOX) in chondrocytes (Lambeth and Neish, 2014). Although physiological ROS levels help maintain cartilage homeostasis, excessive ROS leads to joint oxidative stress, protein carbonylation, DNA damage, and synovial inflammation, aggravating joint damage (Lepetsos and Papavassiliou, 2016; Liu L. et al., 2022). Reduced expression of antioxidant enzymes, including superoxide dismutase (SOD) and glutathione peroxidase (GPx), further worsen oxidative stress. Therefore, the interplay between pro-inflammatory cytokines and excessive ROS creates a self-sustaining cycle that drives cartilage degradation and results in irreversible joint damage if not properly managed.

Cordycepin, a derivative of nucleoside adenosine, is one of the primary bioactive components of *Cordyceps sinensis* (Tuli et al., 2013). Cordycepin exhibits anti-inflammatory (Tan et al., 2021), and immunomodulatory (Zhou et al., 2008) properties, making it an attractive candidate for OA treatment. Studies have shown that cordycepin effectively reduces inflammation through multiple mechanisms (Radhi et al., 2021; Tan et al., 2021). For example, in magnesium silicate-induced osteoporotic rats, cordycepin attenuated inflammation by reducing monocyte infiltration and lowering the levels of inflammatory cytokines such as IL-1β and TNF-α (Zhang et al., 2014). In a Complete Freund’s Adjuvant (CFA)-induced inflammation mouse model, cordycepin inhibited macrophage infiltration and suppressed the production of IFN-γ production (Yang et al., 2020). Additionally, cordycepin significantly alleviated inflammation and asthma-related symptoms by inhibiting key signaling pathways such as NF-κB and p38-MAPK (Yang et al., 2015; Radhi et al., 2021; Tan et al., 2021). Despite its potential, cordycepin undergoes rapid deamination by adenosine deaminase (ADA) into 3′-deoxyadenosine (Tsai et al., 2010), resulting in a short half-life of approximately 1.6 min, which limits its sustained therapeutic efficacy *in vivo*.

Phenylboronic acid (PBA) is a boronic acid derivative with potent antioxidant properties (Ryu et al., 2019). It reduces oxidative stress (Chen et al., 2020; Liu et al., 2020), inflammation (Ryu et al., 2019), and apoptosis in various preclinical models, including OA, by detoxifying ROS and enhancing antioxidant enzyme activity (Ryu et al., 2019). Inflammatory responses in OA joints heavily rely on activated macrophages, which intracellularly produce significant amounts of ROS. Among the various ROS, hydrogen peroxide (H_2_O_2_) is the most stable and abundant, serving as both precursor for other ROS and a mediator of inflammation (Auten and Davis, 2009; Konno et al., 2021; Afzal et al., 2023). Elevated levels of H_2_O_2_ at OA sites exacerbate inflammation by promoting macrophage activation, amplifying pro-inflammatory pathways, and inducing extracellular matrix degradation through lipid peroxidation and DNA damage. Targeting the high levels of ROS, particularly H_2_O_2_, provides a dual therapeutic approach, reducing the oxidative stress and inhibiting macrophage-driven inflammation. PBA can effectively scavenge ROS, including H_2_O_2_, and its anti-inflammatory effects stem from this ROS-scavenging activity. By reducing oxidative stress and inhibiting ROS-mediated inflammatory pathways, PBA attenuates cartilage degradation and slows OA progression. Therefore, PBA could be a promising agent for OA treatment.

Given the complementary mechanisms of cordycepin and PBA, their combination demonstrates the potential as a co-delivered therapeutic strategy against OA. Cordycepin’s anti-inflammatory properties and PBA’s antioxidant effects could enhance cartilage repair and reduce disease progression by simultaneously addressing the key pathological mechanisms of OA. However, their distinct solubility profiles and the need for precise delivery pose significant challenges. A co-delivery system ensures that both compounds reach the target site simultaneously, preserving their combinated ratio and allowing their mechanisms to act in a correct manner. Encapsulation within a single carrier also minimizes premature degradation, especially for cordycepin, reduces dosing frequency, and limits potential side effects.

The polymersome-based system employed in this study could effectively address these challenges (Hasannia et al., 2022; Kansiz and Elcin, 2023). Compared to conventional nanocarriers such as liposomes and micelles, polymersomes demonstrated better stability, controlled drug release, and reduced immunogenicity (Altman, 2004; Bowden et al., 2020; Oo et al., 2021; Qiao et al., 2023). By encapsulating hydrophilic cordycepin and hydrophobic PBA, polymersomes enabled controlled and sustained release of both compounds. These co-delivery polymersomes not only resolved solubility issues but also facilitated interactions between the two compounds, amplifying their combined anti-inflammatory and antioxidant effects.

**SCHEME 1:**
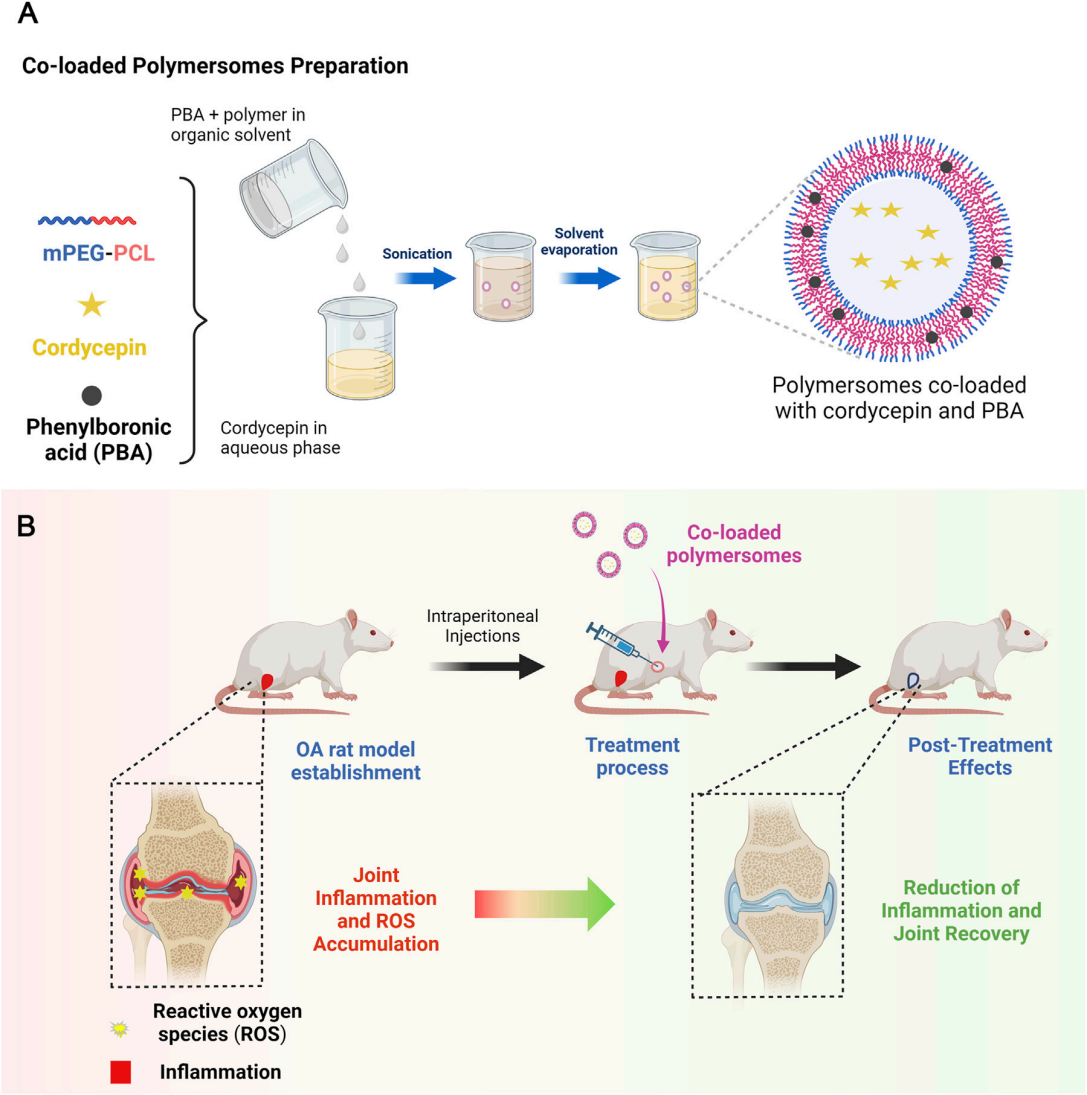
Schematic illustration of co-loaded polymersomes. **(A)** Polymersomes co-loaded with hydrophilic cordycepin and hydrophobic PBA were prepared using an emulsion-solvent evaporation method. **(B)** In the OA rat model, OA rats were treated with co-loaded polymersomes via intraperitoneal (IP) injection every other day for 4 weeks. The combination of anti-inflammatory cordycepin and ROS-scavenging PBA enabled co-loaded polymersomes to effectively reduce pathological cartilage damage and promote cartilage regeneration in OA rats.

In this study, we developed and optimized polymersomes co-loaded with cordycepin and PBA using methoxypoly (ethylene glycol)-b-poly (ε-caprolactone) (mPEG-PCL) (Scheme 1A). This copolymer was chosen for its biocompatibility, biodegradability, and capable of forming stable vesicular structures. The optimization process used a Taguchi orthogonal experimental design to achieve high encapsulation efficiency and a desirable release profile. We evaluated the physicochemical properties of the co-loaded polymersomes *in vitro* and assessed their therapeutic efficacy in a papain-induced OA rat model (Scheme 1B).

## 2 Materials and methods

### 2.1 Materials

All reagents and solvents were obtained from commercial suppliers and used without further purification. Cordycepin, phenylboronic acid (PBA), methoxypoly (ethylene glycol) (mPEG), ε-caprolactone (CL), petroleum ether, polyvinyl alcohol (PVA), high-performance liquid chromatography (HPLC) grade HPLC-grade methanol, HPLC-grade trifluoroacetic acid (TFA), deuterated chloroform (CDCl_3_), and N,N-Diisopropylethylamine (DIPEA) were sourced from China National Pharmaceutical Group Corporation (Shanghai, China). Sulfo-Cyanine7 NHS ester was purchased from Lumiprobe (United States). Additional reagents included Cell Counting Kit-8 (CCK-8) from Dojindo (Japan), Dulbecco’s Modified Eagle Medium (DMEM) from Cytiva HyClone (United States), and fetal bovine serum (FBS), penicillin, and streptomycin from Sijiqing Biologic (China). All materials are explicitly cited with their respective suppliers to ensure reproducibility. Distilled water used in the study was prepared in-house in our laboratory.

### 2.2 Quantification of cordycepin and PBA in co-loaded polymersomes

The determination of cordycepin and PBA was performed by high-performance liquid chromatography (HPLC). To prepare the standard solutions, 5 mg of cordycepin and 10 mg of PBA were dissolved in methanol to form stock solutions of 0.5 mg/mL and 1 mg/mL, respectively. The optimal detection wavelengths for cordycepin and PBA were identified through a full-wavelength UV scan from 200 to 800 nm using methanol as the blank control. The method was established using a Unitary ODS-BP column (250 × 4.6 mm, 5 μm). Isocratic elution was carried out with a mobile phase consisting of 70% methanol and 30% water containing 0.1% trifluoroacetic acid (v/v). The flow rate was 1 mL/min, the column temperature was maintained at 30°C, and the detection wavelength was set to 260 nm. The specificity, linearity, repeatability, stability, and precision of the HPLC method were evaluated using the prepared standard solutions (Supplementary Figures S1–S3; Supplementary Tables S1–S4).

### 2.3 Preparation of polymersomes

#### 2.3.1 Synthesis of methoxypoly (ethylene glycol)-b-poly (ε-caprolactone) (mPEG-PCL)

The synthesis of mPEG-PCL was performed using ring-opening polymerization, using poly (ethylene glycol) (mPEG) as the initiator and stannous octoate as the catalyst. Caprolactone and mPEG were mixed in a polymerization reaction vessel, with the addition of stannous octoate (0.3%, w/w). The synthesis reaction was carried out at 150°C under nitrogen protection for 6 h. The obtained mPEG-PCL was purified through re-precipitation in petroleum ether and dried in a vacuum oven at 40°C. The purified mPEG-PCL was analyzed using nuclear magnetic resonance (NMR) spectroscopy in deuterated chloroform. 1H NMR (400 MHz, CDCl_3_) *δ*, ppm: 1.36 (s, 2H), 1.64 (s, 4H), 2.31 (s, 2H), 3.38 (s, 3H), 3.64 (s, 4H), 4.04 (s, 2H). The spectrum of 1H NMR was presented in Supplementary Figure S4.

#### 2.3.2 Preparation of polymersomes co-loaded with cordycepin and PBA

Co-loaded polymersomes were prepared using an emulsion-solvent evaporation method, which facilitated the encapsulation of both hydrophilic cordycepin and hydrophobic PBA. This method included two phases: emulsification and dilution. During the emulsification phase, mPEG-PCL and PBA were dissolved in 4 mL of chloroform to create the oil phase, while 0.8 mL of an aqueous cordycepin phosphate-buffered saline (PBS) solution (pH 7.4) was added into the mixture. The primary emulsion was formed via ultrasonication at 150 W for 5 min in an ice-water bath. This primary emulsion was then added dropwise to 10 mL of 0.5% polyvinyl alcohol (PVA) solution, followed by ultrasonication at 300 W for 3 additional min. During the dilution phase, the secondary emulsion was gradually added to 40 mL of 0.1% PVA solution under vigorous stirring at 600 rpm for 30 min. Finally, chloroform and excess water were removed using a rotary evaporator to obtain the required polymersomes.

#### 2.3.3 Determination of encapsulation efficiency of co-loaded polymersomes

The purification of co-loaded polymersomes were carried out using a combination of membrane filtration and dialysis. Briefly, an appropriate volume of polymersome solution was first passed through a 0.22 μM membrane to remove large aggregates. The resulting filtrate was transferred into the dialysis tubing (MWCO: 3 kDa) and immersed in 250 mL of PBS, stirred gently for 2 h at room temperature to remove free cordycepin. After dialysis, the sample was centrifuged at 10,000 rpm for 10 min to remove any precipitated PBA or particulate residues. The obtained polymersomes were freeze-dried for future use.

To determine encapsulation efficiency (EE%) and drug loading efficiency (DL%), 5 mg of lyophilized drug-loaded polymersomes were dispersed in 5 mL DI water. The solution was mixed with methanol and sonicated to ensure complete release of encapsulated compounds. The concentrations of cordycepin and PBA were then determined using the HPLC method described above. EE% and DL% were calculated as follows:

$$\text{Encapsulation efficiency }\left(\text{EE}\%\right)=\frac{W_{\text{encapsulated}\;\text{compund}}}{W_{\text{feeding}\;\text{compound}}}\times 100\%$$

$$\text{Drug loading efficiency }\left(\text{DL}\%\right)=\frac{W_{encapsulated\;compound}}{W_{total\;polymersome}}\times 100\%$$

where *W*_encapsulated compound_ represented the weight of the compound encapsulated in the loaded polymersomes, *W*_feeding compound_ represented the total weight of the feeding compound, and *W*_total polymersomes_ represented the total weight of the compound-loaded polymersomes.

### 2.4 Formulation optimization

The optimization of polymersome formulation was conducted to enhance the encapsulation efficiency of both cordycepin and PBA. A systematic approach was used, involving One-Factor-At-A-Time (OFAT) experiments and a Taguchi orthogonal experimental design (Ballantyne et al., 2008; Krishnaiah and Shahabudeen, 2012).

#### 2.4.1 OFAT experiment

OFAT experiments were carried out to investigate the effects of various variables on encapsulation efficiency, including ultrasonic power for primary emulsification, ultrasonic power for secondary emulsification, PVA concentration in the aqueous phase, organic-to-aqueous phase ratio, and emulsification stirring speed. During each OFAT experiment, all variables were held constant except for the parameter under investigation. The ultrasonic power for primary emulsification was varied at 100 W, 150 W, 200 W, while the secondary emulsification power was varied from 150 W to 300 W in increments of 50 W. PVA concentration in the aqueous phase was varied from 0.5% to 2%, the organic-to-aqueous phase ratio varied between 1:4 and 1:7, and the emulsification stirring speed ranged from 200 rpm to 800 rpm.

#### 2.4.2 Orthogonal experiment

Based on the significant parameters identified from the OFAT experiments, a Taguchi orthogonal experimental design was applied to further optimize the formulation. Selected factors were evaluated using a L_9_ (3^4^) orthogonal array, which involved 9 experiments with three levels across four factors. Encapsulation efficacy was selected as the response variable, and the results were analyzed statistically using range analysis. Subsequently, optimal polymersomes were prepared under the best conditions to investigate the maximum encapsulation efficiency.

### 2.5 Characterization of co-loaded polymersomes

The *in vitro* characterization of the co-loaded polymersomes was performed to assess their potential as therapeutic agents for OA, particularly in a microenvironment with high levels of ROS. Characterization included morphological analysis, particle size distribution, stability assessment, and *in vitro* drug release studies.

#### 2.5.1 Particle size and zeta potential

The particle size and polydispersity index (PDI) were determined using dynamic light scattering (Nano-ZS 90, Malvern). Measurements were carried out in triplicate at 25°C after equilibration for 2 min.

#### 2.5.2 Stability

The stability of the polymersomes was evaluated by monitoring changes in particle size distribution and PDI under 4°C over a 1-month period. Observations were recorded at intervals of 0, 10, 20, and 30 days.

#### 2.5.3 Morphological analysis

For morphological analysis, co-loaded polymersomes were placed onto a specialized copper mesh, negatively stained with 2% phosphotungstic acid, and then dried at room temperature. The morphology of the co-loaded polymersomes was observed under a transmission electron microscopy (TEM).

#### 2.5.4 X-ray diffraction (XRD) study

XRD analysis was carried out to determine the crystallographic structure of the co-loaded polymersomes. The patterns of free compound combination, blank polymersomes, a physical mixture of free compound combination and blank polymersomes, and co-loaded polymersomes were analyzed using an X-ray diffractometer with Cu Ká radiation. Measurements were performed at a voltage of 40 kV and a tubing current of 30 mA. The scanned angle range was set at 2θ from 10° to 80° with the scanning speed of 0.02°/min.

#### 2.5.5 *In vitro* release profiles

The *in vitro* release profile of co-loaded polymersomes was determined using the dialysis method. Briefly, co-loaded polymersomes were placed in dialysis bags (MWCO = 3 kDa) and incubated in 50 mL of PBS buffer containing varying concentrations of H_2_O_2_ (0, 50, and 100 µM) to mimic the osteoarthritic environment. The incubation was carried out at 37°C with gentle shaking. Aliquots were taken at predetermined time points, replaced with fresh media, and stored at −20°C for further analysis. After dialysis, the concentrations of cordycepin and PBA were determined using the HPLC method as described above.

#### 2.5.6 *In vitro* ROS scavenging test

The ROS scavenging ability of co-loaded polymersomes was assessed using luminol-based chemiluminescence (Khan et al., 2014). Briefly, a series of co-loaded polymersome solutions with varying concentrations of PBA (0, 10, 100, 250, 500, and 750 μg/mL) were prepared. In each well of a white 96-well microplate, 100 μL of H_2_O_2_ solution (100 μM) was mixed with 100 μL of each polymersome solution. Then, 10 μL of CuCl_2_ solution (100 μM) and 50 μL of luminol solution (50 μM) were added in sequence. After thorough mixing, the chemiluminescence intensity was measured using a microplate reader. The ROS scavenging efficiency of the co-loaded polymersomes at a concentration of *x* μg/mL was calculated using the following equation:

$${Scavening\;efficiency}_{x\;\mu g/mL}\left(\%\right)=\frac{{{Intensity}_{0\;\mu g/mL}-Intensity}_{x\;\mu g/mL}}{{Intensity}_{0\;\mu g/mL}}\times 100$$

### 2.6 *In vitro* cellular assays

#### 2.6.1 Cell culture

Murine RAW264.7 macrophage cell line was purchased from the Cell Bank of Type Culture Collection, Chinese Academy of Science (Shanghai, China). Cells were cultured in DMEM supplemented with 10% FBS and 1% penicillin/streptomycin under the condition of 37°C and 5% CO_2_.

#### 2.6.2 Cell viability assay

The cytotoxicity of free compounds, their combination, and co-loaded polymersomes against RAW264.7 cells were assessed using the CCK-8 assay. Briefly, cells were seeded into 96-well plates at a density of 2 × 10^4^ cells/well and incubated overnight. Subsequently, cells were treated with varying sample concentrations for 48 h. For the combination group and co-loaded polymersomes, cordycepin and PBA were mixed in equal or near-equal molar ratios, with each compound contributing approximately half of the total concentration. Subsequently, 10 μL of CCK-8 solution was added into each well and incubated for another 2 h. Finally, absorbance was measured at 490 nm using a microplate reader (BioTek XS2, United States).

#### 2.6.3 Cytokine analysis

RAW264.7 cells were seeded in 24-well plate at a density of 1× 10^5^ cells/well. After overnight culture, cells were pre-treated with lipopolysaccharide (LPS, 1 μg/mL) for 12 h to induce an inflammatory response. Subsequently, cells were exposed to free cordycepin, free PBA, their free compound combination, or co-loaded polymersomes for 24 h. Cytokine contents, including IL-1β and TNF-α, in the supernatants were determined using commercially available ELISA kits (LianKe Bio Co., Ltd., Hangzhou, China) according to the manufacturer’s instructions. Absorbance was then measured at 520 nm using a microplate reader.

#### 2.6.4 Measurement of ROS production

ROS production in RAW264.77 cells were measured using the Amplex Red hydrogen peroxide assay (Mishin et al., 2010; Karakuzu et al., 2019). This assay employs horseradish peroxidase (HRP) to catalyze the oxidation of 10-acetyl-3,7- dihydroxypenoxazine in the presence of H_2_O_2_, resulting in the red fluorescent product resorufin. In brief, cells were seeded in 96-well plate with phenol-free complete DMEM medium, treated with LPS, and subsequent treated with various samples. Then, 50 μL of culture supernatants were mixed with 50 μL of Amplex Red working buffer containing 50 μM Amplex Red and 10 U/mL HRP. The mixture was incubated in the dark at room temperature for 30 min. The fluorescence was detected with an excitation wavelength of 530 nm and an emission wavelength of 590 nm in a microplate reader.

### 2.7 *In vivo* studies

#### 2.7.1 Animals

Specific pathogen-free (SPF) grade Sprague Dawley (SD) rats (weight 200 ± 20 g, 50 females) were purchased from Jiangsu University. All rats were maintained in a temperature-controlled facility with a 12-h light/dark cycle at 24°C and provided free access to standard diet and tap water. All experimental procedures were ethically approved by the Animal Use and Care Committee of Jiangsu University (Approval number: 11,657), and all procedures were conducted in accordance with the NIH guidelines for the Care and Use of Laboratory Animals.

#### 2.7.2 The establishment of OA rat model

The OA rat model was established according to the reference (Wang et al., 2014; Cheng et al., 2019; Liu et al., 2021). A solution of 5% papain and 0.03 M L-cysteine was mixed at a volume ratio of 1:2, where L-cysteine served to preserve the activity of papain. The obtained mixture (10 μL) was injected into the left knee joint of the rats on days 0, 4 and 7 at the same dose. Rats in the control group were injected with equal volumes of sterile saline in the left knee joint. Two weeks post-injection, the OA rat model was successfully established. Prior to treatments, the diameters of right and left knee were determined every other day in the medial-lateral direction, with the caliper positioned perpendicular to the leg axis. The joint swelling was expressed as the ratio of the knee diameter to the baseline mean knee diameter.

#### 2.7.3 *In vivo* imaging of co-loaded polymersomes following intraperitoneal (IP) injection

To investigate the biodistribution, co-loaded polymersomes were labeled with the water-soluble near-infrared dye sulfo-Cyanine7 (sulfo-Cy7). In brief, 1 μmol of sulfo-Cy7 NHS ester was dissolved in 1 mL of anhydrous DMF, and 1.1 equivalents of mPEG-PCL-NH_2_ and DIPEA were added. The mixture was stirred in the dark for 1 h at room temperature, followed by dialysis in the dark to remove the organic solvent. Finally, the Cy7-labeled polymers were incorporated into polymersomes at the percentage of 0.5% during preparation.

Cy7-labeled co-loaded polymersomes were then injected intraperitoneally into OA rats. The rats were anaesthetized with 2% isoflurance and whole-body images were captured after administration using the Xtreme *In vivo* Imaging System at 0, 12 and 24 h. For imaging Cy7, an excitation filter of 750 nm and an emission filter of 830 nm were used. Additionally, images of various organs were also captured after sacrifice of the OA rat 24 h post-administration.

#### 2.7.4 *In vivo* efficacy studies

OA rats were randomly divided into six groups (n = 7) and treated intraperitoneally (IP) every other day for 4 weeks with: (1) saline, (2) free cordycepin (15 mg/kg), (3) free PBA (7.5 mg/kg), (4) free combination of cordycepin and PBA (15 mg/kg cordycepin and 7.5 mg/kg PBA), (5) co-loaded polymersomes (15 mg/kg cordycepin and 7.5 mg/kg PBA), and (6) blank polymersomes. Healthy rats were used as control. The IP injection volume was kept at 0.75 mL. Periodic measurements of body weights and joint swelling of rats were performed throughout the study.

After 4 weeks of treatment, rats were euthanized, and sacrificed for further analysis. Serum levels of IL-1β and TNF-α were detected using ELISA kits (Lianke Bio, Hangzhou, China).

#### 2.7.5 Histological preparation

All collected knee joints were fixed in 10% formalin for 3 days, decalcified for 5 days, embedded in paraffin, and sectioned into 5-μm thick slices with 200-μm intervals. The paraffin sections were dewaxed, hydrated in graded alcohol, and stained using hematoxylin and eosin (H&E) solution. The pathological changes in the articular cartilage were analyzed using Image-Pro image analysis software.

### 2.8 Statistical analysis

All statistical analyses were performed using GraphPad Prism version 6.01 (San Diego, CA, United States). Differences were determined using Student’s t-test or one-way ANOVA followed by Tukey’s *post hoc* tests. A *p-*values less than 0.05 was considered statistically significant. Data were presented as mean ± standard deviation (SD).

## 3 Results

### 3.1 Preparation and optimization of co-loaded polymersomes

We synthesized the copolymer mPEG2k-PCL5k at an optimal feed ratio of mPEG2k to ε-caprolactone (CL). The structure of mPEG2k-PCL5k was confirmed using 1H NMR, as shown in Supplementary Figure S1. Co-loaded polymersomes were prepared using an emulsion-solvent evaporation method, and the formulation was systematically optimized. To determine the factors influencing the preparation of co-loaded polymersomes, we performed one-factor-at-a-time (OFAT) experiments, investigating the effects of various factors on encapsulation efficiency for both cordycepin and PBA.

#### 3.1.1 OFAT experiments

**FIGURE 1:**
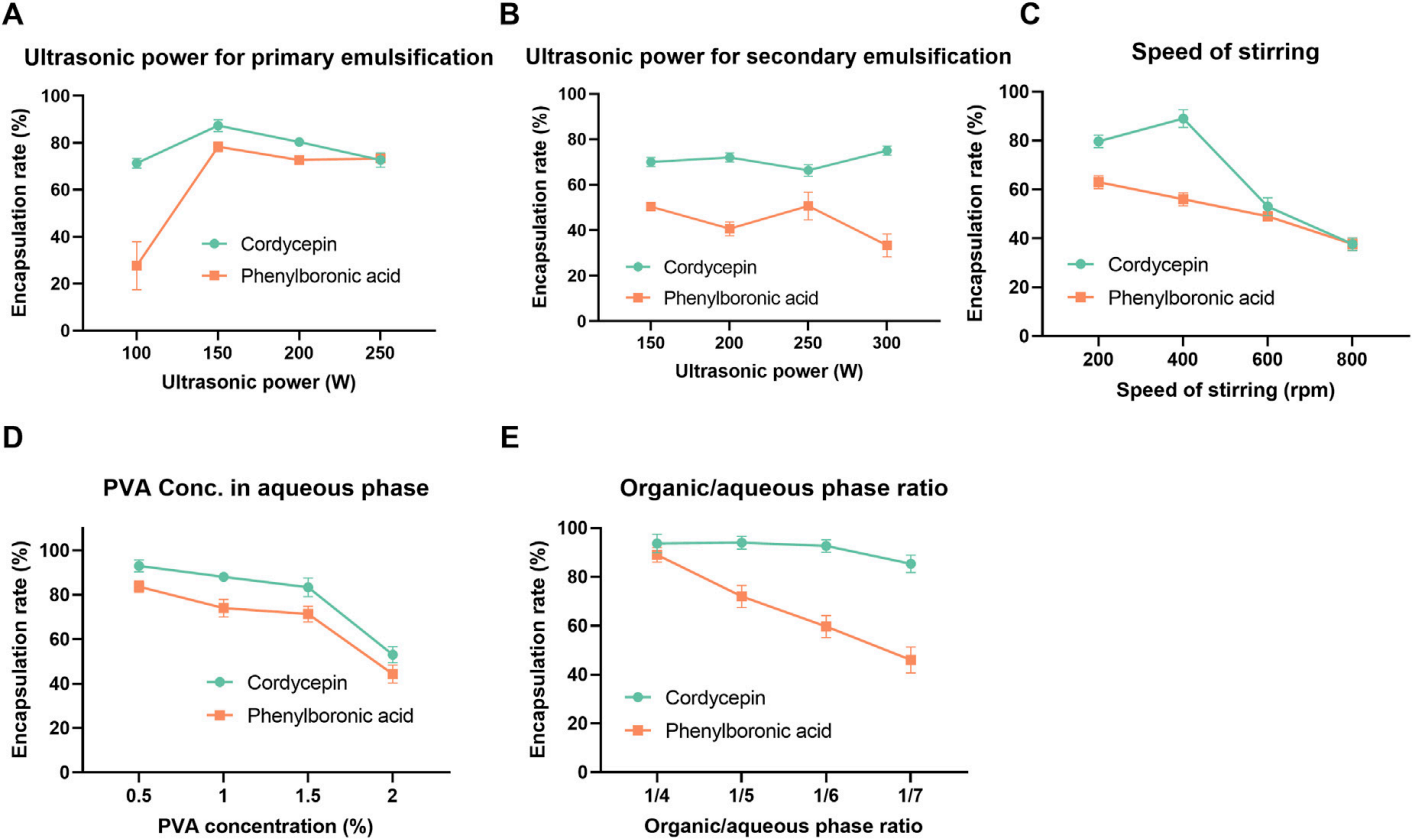
One-factor-at-a-time (OFAT) optimization results of co-loaded polymersomes. The effects of various process and formulation parameters, including ultrasonic power for primary **(A)** and secondary emulsification **(B)**, stirring speed **(C)**, PVA concentration in the aqueous phase **(D)**, and organic/aqueous phase ratio **(E)**, on the encapsulation efficiency of cordycepin and PBA. Data are mean ± SD (n = 3).

Due to the distinct physicochemical properties, cordycepin and PBA exhibited different encapsulation trends. The encapsulation efficiency peaked at an optimal sonication power of 150 W for primary emulsification (Figure 1A). In detail, at lower sonication powers, phase separation within the vesicle material was observed, resulting in reduced encapsulation efficiency. Beyond the optimal level, further increases in sonication power led to a decline in encapsulation efficiency, which may be associated with disruption of the vesicle structure due to excessive ultrasonic energy (Yamaguchi et al., 2009; Yang et al., 2021). However, secondary emulsification power had a minimal effect on encapsulation efficiency (Figure 1B). Stirring speed during emulsification significantly impacted encapsulation; increasing speed improved encapsulation until a threshold was reached, beyond which encapsulation efficiency declined (Figure 1C). Stirring speed had a stronger influence on the encapsulation efficiency of cordycepin than PBA, likely due to differences in their solubility and molecular interactions with the polymer membranes.

Formulation factors were also investigated. As shown in Figure 1D, cordycepin exhibited a marked decrease in encapsulation efficiency when the PVA concentration increased from 1.5% to 2%, suggesting that higher PVA content may interfere with effective entrapment. In contrast, PBA showed a slight increase in encapsulation efficiency between 1% and 1.5% PVA, followed by a minor decline at 2%, indicating that intermediate PVA concentrations are more favorable for PBA loading. Additionally, increasing the organic-to-aqueous phase ratio slightly decreased cordycepin encapsulation efficiency while significantly reducing PBA encapsulation efficiency, dropping from 86% to approximately 50% (Figure 1E). This decline was attributed to the instability of the primary emulsion. When the volume of the aqueous phase increased as the organic-to-aqueous phase ratio reduced from 1/4 to 1/7, the size of the resulting primary emulsion significantly increased, compromising its structural integrity. This loss of stability eventually led to the breakdown of the emulsion and the subsequent release of PBA from the organic layer. Overall, PBA exhibited consistently lower encapsulation efficiency than cordycepin during the OFAT optimization.

#### 3.1.2 Taguchi orthogonal experimental optimization

The results of OFAT experiments indicated that secondary emulsification power had minimal influence on encapsulation efficiency, so four other factors were selected for further optimization using a Taguchi orthogonal design. Based on the OFAT results, the ultrasonic power for primary emulsification was evaluated between 100 W and 200 W, while PVA concentration was examined between 0.5% and 1.5%. The organic/aqueous phase ratio was tested at 1:4, 1:5, and 1:6. Lastly, stirring speed was set at 200, 400, and 600 rpm.

An *L*_9_ (3^4^) Taguchi orthogonal design was applied to identify the optimal preparation conditions for maximizing the encapsulation efficiency of cordycepin and PBA. After 9 experimental runs, the results were presented in Table 1, 2. We analyzed the effects of each factor level on the encapsulation efficiency for cordycepin and PBA using range analysis. For cordycepin, the key factors influencing encapsulation efficiency were ranked as follows: PVA concentration in the aqueous phase (B_1_, 0.5%) > ultrasonic power (A_1_, 100 W) > stirring speed (C_3_, 800 rpm) > organic/aqueous phase ratio (D_1_, 1:4). Similarly, the factors influencing PBA encapsulation were ranked, with a slight variation: ultrasonic power (A_1_, 100 W) > PVA concentration (B_1_, 0.5%) > organic/aqueous phase ratio (D_1_, 1:4) > stirring speed (C_3_, 800 rpm).

**TABLE 1:** Taguchi orthogonal experiment results for cordycepin encapsulation efficiency.

NO.	A (ultrasonic power)	B (PVA conc. in aqueous phase)	C (stirring speed)	D (organic/aqueous phase ratio)	Cordycepin EE%
1	100	0.5%	400	1:4	98
2	100	1.0%	600	1:5	91
3	100	1.5%	800	1:6	95
4	150	0.5%	600	1:6	97
5	150	1.0%	800	1:4	95
6	150	1.5%	400	1:5	89
7	200	0.5%	800	1:5	97
8	200	1.0%	400	1:6	39
9	200	1.5%	600	1:4	85
K1	284.000	292.000	226.000	278.000	
K2	281.000	225.000	273.000	277.000	
K3	221.000	269.000	287.000	231.000	
R	21.000	22.333	20.333	15.667	

**TABLE 2:** Taguchi orthogonal experiment results for PBA encapsulation efficiency.

NO.	A (ultrasonic power)	B (PVA conc. in aqueous phase)	C (stirring speed)	D (organic/aqueous phase ratio)	PBA EE%
1	100	0.5%	400	1:4	87
2	100	1.0%	600	1:5	67
3	100	1.5%	800	1:6	81
4	150	0.5%	600	1:6	65
5	150	1.0%	800	1:4	75
6	150	1.5%	400	1:5	76
7	200	0.5%	800	1:5	71
8	200	1.0%	400	1:6	40
9	200	1.5%	600	1:4	62
K1	235.000	223.000	203.000	224.000	
K2	216.000	182.000	194.000	214.000	
K3	173.000	219.000	227.000	186.000	
R	20.667	13.667	11.000	12.667	

The observed differences in factor rankings for the two compounds required a strategic compromise to optimize both encapsulation efficiencies simultaneously. Consequently, we prioritized maximizing cordycepin encapsulation while ensuring high PBA encapsulation. Based on the Taguchi experimental design, the optimal formulation conditions were determined to be a PVA concentration in aqueous phase of 0.5%, ultrasonic power of 100 W, stirring speed of 800 rpm, and an organic/aqueous phase ratio of 1:4.

#### 3.1.3 Confirmation of Taguchi optimization

The confirmation experiments validated the optimal formulation conditions. As shown in Table 3, the experiment achieved encapsulation efficiencies of 97.61% ± 0.40% for cordycepin and 86.12% ± 0.95% for PBA. In terms of drug loading efficiency, the optimized formulation showed a DL% of 4.47 ± 0.02 for cordycepin and 3.95 ± 0.04 for PBA. The result demonstrated the reproducibility of the optimized formulation, further confirming its suitability for co-loaded application.

**TABLE 3:** Confirmation results for Taguchi orthogonal experiment optimization (n = 3).

Formulation type	Cordycepin EE%	PBA EE%	Cordycepin DL%	PBA DL%	Particle size (nm)	PDI	Zeta potential (mV)
Blank polymersomes	–	–	–	–	87.9 ± 3.6	0.153 ± 0.012	−11.0 ± 1.5
Co-loaded polymersomes	97.61 ± 0.40	86.12 ± 0.95	4.47 ± 0.02	3.95 ± 0.04	101.03 ± 0.42	0.248 ± 0.014	−9.4 ± 1.3

### 3.2 Characterization of co-loaded polymersomes

#### 3.2.1 Size, size distribution and morphology

**FIGURE 2:**
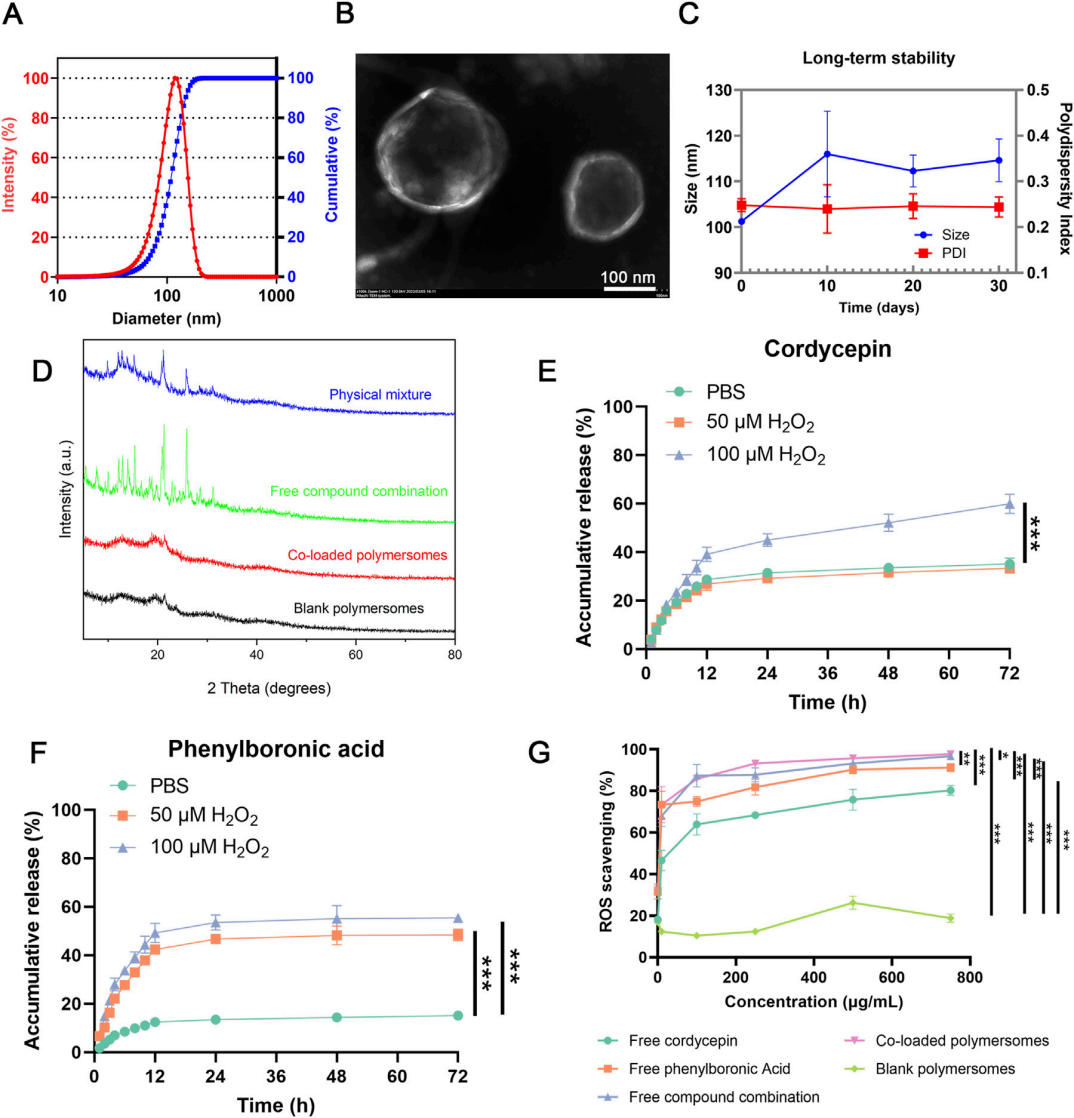
*In vitro* characterization of co-loaded polymersomes. **(A)** Size and size distribution of co-loaded polymersomes. **(B)** Representative TEM image of co-loaded polymersomes, displaying typical spherical morphology and uniform size. Scale bar: 100 nm. **(C)** Long-term stability of co-loaded polymersomes at 4°C for over 1 month (n = 3). **(D)** XRD analyses of different samples. **(E,F)** *In vitro* release profiles of cordycepin and PBA from co-loaded polymersomes under different H_2_O_2_ concentrations. Data are mean ± SD (n = 3). **(G)** ROS scavenging activities of various samples at different concentrations. *p < 0.05, **p < 0.005, and ***p < 0.001 versus other groups. Data are mean ± SD (n = 3).

As shown in Figure 2A, DLS measurements revealed that the optimal co-loaded polymersomes had an average size of 101.03 ± 0.42 nm with a polydispersity index (PDI) of 0.248 ± 0.014, indicating a relatively narrow size distribution. TEM images further confirmed that co-loaded polymersomes were relatively spherical in shape, with sizes consistent with the DLS data (Figure 2B).

Additionally, the co-loaded polymersomes exhibited a relatively high colloidal stability and could maintain their size and size distribution in PBS at 4°C for over 1 month, as shown in Figure 2C. At the end of the 30-day stability study, the encapsulation efficiencies decreased to 80.53% ± 3.63% for cordycepin and 65.43% ± 8.72% for PBA, indicating moderate drug retention under 4°C.

#### 3.2.2 X-ray diffraction (XRD) analysis of co-loaded polymersomes

The crystalline form of the encapsulated compounds could affect their therapeutic efficacy. XRD analysis was performed to evaluate the polycrystalline structure of the co-loaded polymersomes. As shown in Figure 2D, the combination of free cordycepin and PBA exhibited multiple crystal diffraction peaks, indicating their crystalline states.

In contrast, blank polymersomes had no obvious characteristic peaks, consistent with an amorphous structure. Notably, the XRD pattern of the co-loaded polymersomes demonstrated that the characteristic diffraction peaks of cordycepin and PBA were nearly eliminated, suggesting that both compounds existed in an amorphous form within the polymersomes (Thakral et al., 2016). This transition to an amorphous state likely enhance their encapsulation and stability, demonstrating the effectiveness of the polymersomes in preventing crystallization.

#### 3.2.3 *In vitro* release profile

The *in vitro* release profiles of cordycepin and PBA from the polymersomes were investigated under varying H_2_O_2_ concentrations. The H_2_O_2_ concentrations selected for the release study (0, 50, and 100 µM) were used to simulate both physiological (baseline) and pathological (inflamed) conditions of the OA microenvironment, indicating that synovial fluid in osteoarthritic joints exhibits elevated oxidative stress (Zhang et al., 2021; Jiang et al., 2022; Wang et al., 2023). As shown in Figure 2E, approximately 35% of cordycepin was released after 72 h under 0 μM or 50 μM H_2_O_2_. In contrast, at 100 μM H_2_O_2_, the cumulative release rose significantly to 59%. Similarly, PBA exhibited a slower release profile, with less than 20% released after 72 h in PBS alone (Figure 2F). Under oxidative conditions of 50 μM and 100 μM H_2_O_2_ for 72 h, the cumulative release of PBA increased to 48% and 55%, respectively. These results indicated that the presence of H_2_O_2_ could significantly accelerate the release of both compounds from the polymersomes in a concentration-dependent manner.

#### 3.2.4 ROS-scavenging ability

The ROS scavenging ability of the co-loaded polymersomes was evaluated using a chemiluminescence assay in a high ROS environment. In this study, H_2_O_2_ was used to mimic a high-ROS environment, with the H_2_O_2_-Cu^2+^ reaction generating hydroxyl radicals (·OH). Subsequently, luminol-based chemiluminescence was employed to measure the scavenging effect of the compounds on hydrogen peroxide.

As shown in Figure 2G, the *in vitro* ROS scavenging capability increased with compound concentration. Free PBA exhibited greater ROS scavenging activity compared to free cordycepin, and their combination demonstrated enhanced activity. Notably, the co-loaded polymersomes exhibited the highest scavenging efficacy, reaching up to 97% at a concentration of 750 μM, indicating a strong dose-dependent response. In contrast, blank polymersomes showed minimal ROS scavenging ability across the tested concentration range. These results confirmed that the co-loaded polymersomes exhibited ROS scavenging efficacy comparable to that of the free compound combination, effectively reducing ROS levels at the site of OA.

### 3.3 Cellular assay

#### 3.3.1 Cell viability

**FIGURE 3:**
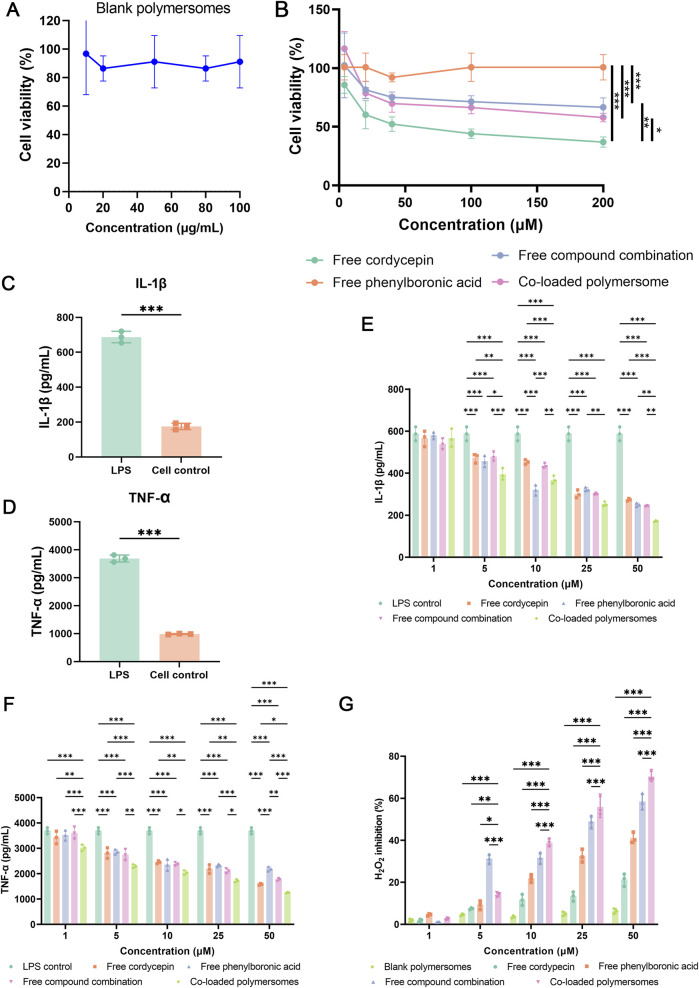
Cytotoxicity, anti-inflammatory effects, and oxidative stress regulation of co-loaded polymersomes in RAW 264.7 macrophages. **(A,B)** Cytotoxicity evaluation of blank polymersomes and various formulations. Cell viability of RAW 264.7 macrophages was assessed after incubation with blank polymersomes **(A)** and different treatments at varying concentrations **(B)**. Each treatment group represents either single compounds or their combination at equivalent total concentrations. For the combination group and co-loaded polymersomes, cordycepin and PBA were administered at equal or near-equal molar ratios. **(C–F)** Anti-inflammatory effects of different treatment groups on LPS-induced RAW 264.7 macrophages. The secretion levels of IL-1β **(C)** and TNF-α **(D)** were quantified following LPS stimulation, while the inhibitory effects of various samples on IL-1β **(E)** and TNF-α **(F)** secretion were analyzed. *p < 0.05, **p < 0.005, and ***p < 0.001 versus other groups. Data are presented as mean ± SD. **(G)** Measurement of H_2_O_2_ production in RAW 264.7 macrophages using the Amplex Red assay. Cells were stimulated with LPS and treated with different formulations for 24 h. Data are presented as mean ± SD (n = 3). *p < 0.05, **p < 0.01, and ***p < 0.001 compared with the co-loaded polymersomes group.

The cytotoxicity of different groups on RAW264.7 cells were evaluated using the CCK-8 assay. As shown in Figure 3A, blank polymersomes had negligible effects on cell viability, confirming their biocompatibility. Among the treatments, free cordycepin exhibited the highest cytotoxicity, likely due to its ability to suppress NF-κB signaling pathway (Huang et al., 2021), with an IC_50_ value of 101.4 ± 18.96 μM. In contrast, free PBA exhibited relatively low cytotoxicity, indicating a favorable safety profile. The free compound combination revealed intermediate cytotoxicity between free cordycepin and free PBA. Co-loaded polymersomes exhibited similar cytotoxicity to the free compound combination in RAW 264.7 cells across all concentrations tested (Figure 3B). These results suggest that the encapsulated formulation maintains the biocompatibility profile of the free combination and may reduce the direct exposure of cells to free cordycepin at high local concentrations.

#### 3.3.2 Effect of co-loaded polymeromes on pro-inflammatory cytokine production in LPS-induced RAW264.7 macrophages

To investigate the anti-inflammatory effects of various samples, RAW264.7 cells were pre-treated with LPS to simulate an inflammatory environment of OA. As shown in Figures 3C,E, the exposure of LPS for 24 h resulted in a significant increase in the secretion of pro-inflammatory cytokines, including IL-1β and TNF-α, from RAW264.7 cells. Treatment with various groups prior to LPS activation could attenuate the release of these pro-inflammatory cytokines in a dose-dependent manner (Figures 3D,F). The free compound combination at 50 μM exhibited greater inhibition of cytokines production compared to either compound alone. Furthermore, at the same concentration (50 μM), the co-loaded polymersomes exhibited inhibitory effects on cytokine secretion that were statistically comparable to those of the free compound combination. This result may be attributed to the co-delivery and protective properties of the polymersomes, which help maintain the stability and localized availability of both compounds.

#### 3.3.3 Antioxidant effect of co-loaded polymersomes on RAW264.7 cells

Unlike the luminol assay, the Amplex Red method cannot penetrate the cell membrane. Therefore, this method was used to determine the concentration of H_2_O_2_ in the cell supernatant following various treatments to assess the antioxidant effects of these treatments.

As shown in Figure 3G, blank polymersomes exhibited negligible antioxidant activity at the test concentrations. In contrast, free compounds and co-loaded polymersomes demonstrated dose-dependent antioxidant activity. Free PBA showed superior efficacy in reducing H_2_O_2_ production in RAW264.7 cells compared to free cordycepin. The combination of both compounds exhibited significantly higher antioxidant activity than either free compound alone. Notably, co-loaded polymersomes demonstrated the greatest inhibition of H_2_O_2_ production at higher concentration compared to the free compound combination. The improved inhibitory effect of co-loaded polymersomes is likely due to their sustained release properties, where provided prolonged antioxidant effects.

### 3.4 *In vivo* animal model

#### 3.4.1 Biodistribution of co-loaded polymersomes in OA rats

**FIGURE 4:**
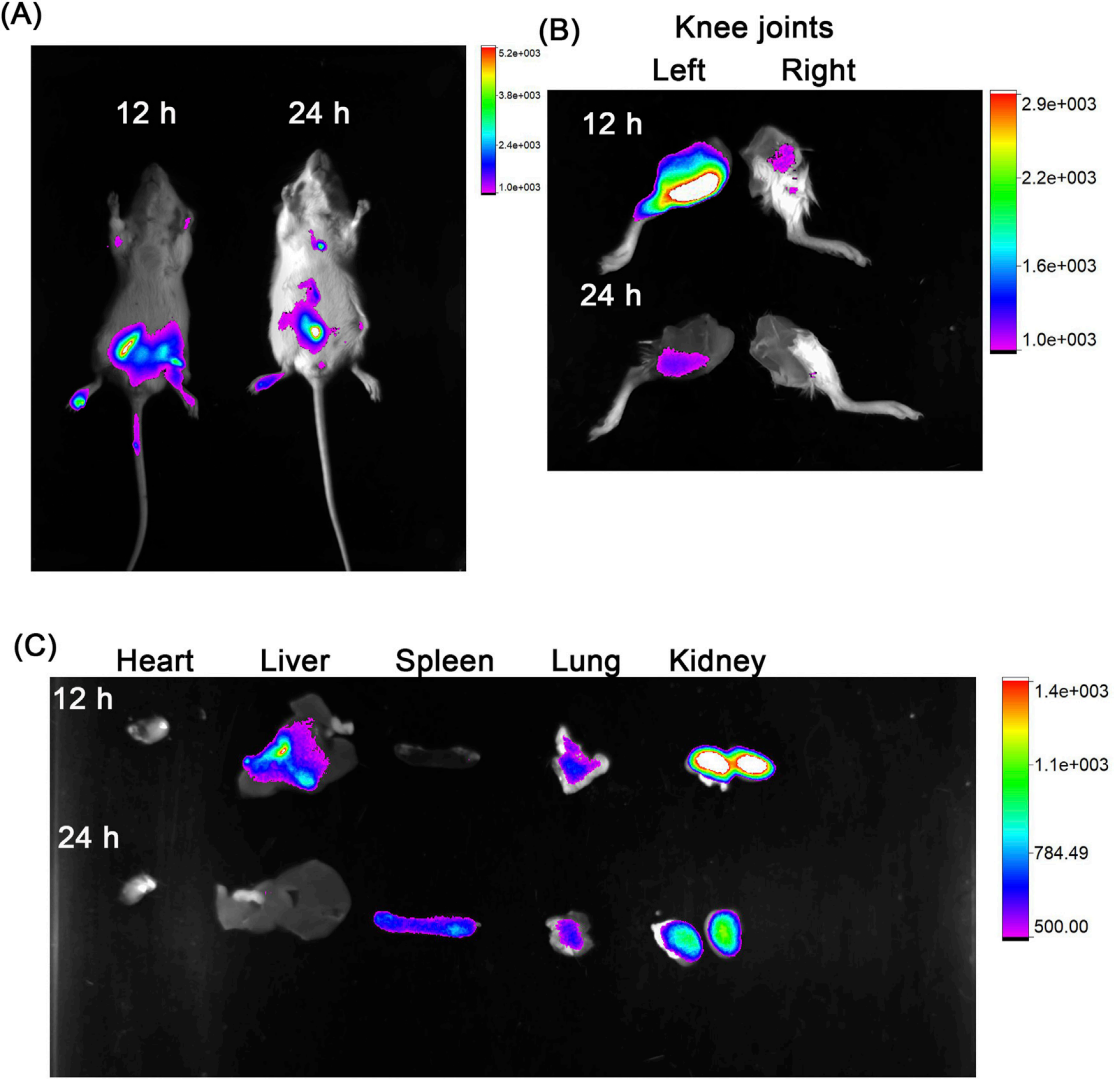
*In vivo* targeting capacity of co-loaded polymersomes in an OA rat model. **(A)** OA rats, with induced OA in the left knee joint and an unaffected right knee joint considered healthy, were imaged at 12 h and 24 h post-intraperitoneal (IP) injection using a Bruker small animal *in vivo* imaging system. **(B)** Imaging of the knee joints at 12 h and 24 h post-IP injection, showing the inflamed left knee and healthy right knee. **(C)** Imaging of major organs, including heart, liver, spleen, lung, and kidney, at 12 h and 24 h post-IP injection, showing the distribution of co-loaded polymersomes across different tissues.

The *in vitro* activity of the co-loaded polymersomes allowed us to investigate their therapeutic effects in an OA rat model. We first assessed the targeting potential of the co-loaded polymersomes using Cy7-labeled polymersomes. These polymersomes were administered via intraperitoneal (IP) injected in OA rats, with induced OA in the left knee joint and an unaffected right knee joint serving as the healthy control. As shown in Figure 4A, Cy7 fluorescence signals were detected in the inflamed left knee joint at 12 h post-injection, with signals persisting for up to 24 h. In contrast, minimal fluorescence was observed in the right, healthy knee joint at both time points. This result clearly demonstrated the ability of the co-loaded polymersomes to target the OA-affected site. The rats were subsequently sacrificed for *ex vivo* imaging of the knee joint and major organs. As shown in Figure 4B, the left knee joint exhibited strong Cy7 fluorescence signals at 12 h post-injection, which weakened at 24 h. To further investigate the distribution of co-loaded polymersomes in major organs, these tissues were harvested at 12 h and 24 h post-injections (Figure 4C). The Cy7 signals were detected in the liver and kidney at 12 h post-injection, but the signal intensity diminished at 24 h post-injection. These results indicated the co-loaded polymersomes efficiently targeted the OA site and accumulated in the inflamed joint.

#### 3.4.2 Joint swelling in OA rats treated with co-loaded polymersomes

To evaluate the therapeutic effects of the treatment on joint swelling (edema), changes in joint width and body weight were measured.

**FIGURE 5:**
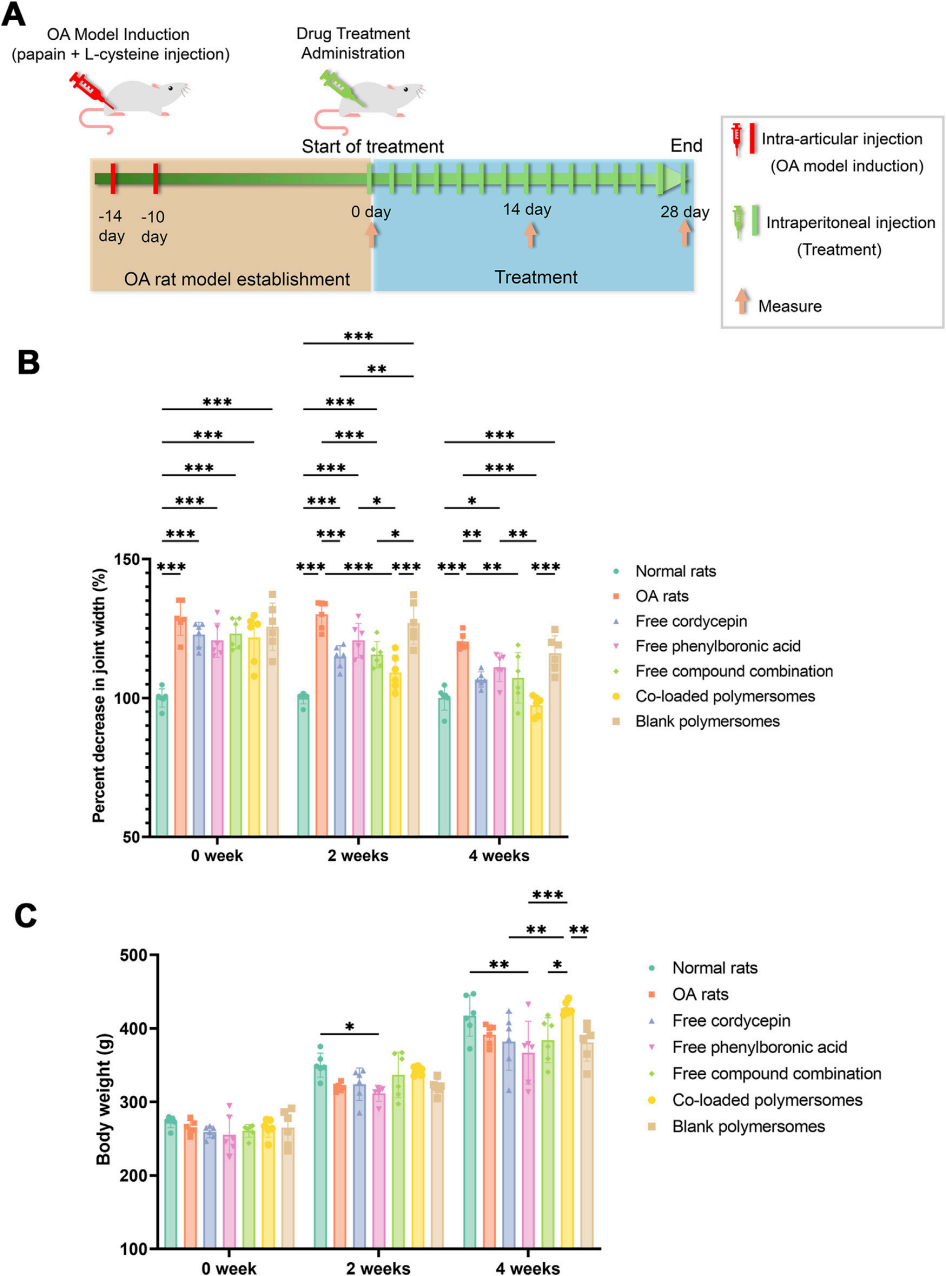
Joint swelling of OA rats after 4-week treatments with different groups. **(A)** Schematic representation of the OA induction and administration protocol. **(B)** Percentage decrease in joint width of OA rats at days 14 and 28. **(C)** Changes in body weights of OA rats at days 14 and 28. *p < 0.05, **p < 0.01, and ***p < 0.001 compared with OA model rats (n = 7).

As shown in Figure 5A, OA was induced via intra-articular injection of papain and L-cysteine on days −14 and −10, followed by intraperitoneal administration of different treatment formulations from day 0 to day 28. Key experimental time points for measurements are highlighted, including joint swelling and body weight assessments at days 14 and 28.

As a result, Figure 5B indicated that a significant increase in joint width was observed in OA rats compared to normal rats prior to treatment, indicating that the injection of papain and L-cysteine successfully induced joint inflammation in the rats. Compared with untreated OA rats, various treatments reduced the joint width in OA rats over the 4-week study period. At 2 weeks post-administration, rats treated with co-loaded polymersomes showed an 11.45% ± 4.31% reduction in joint width, compared to a 7.76% ± 2.59% reduction in rats treated with the free compound combination. At 4 weeks post-administration, the reduction in joint width was 19.17% ± 3.58% in the co-loaded polymersome group, compared to 9.31% ± 2.00% for the free compound combination group. These results indicated that joint width for co-loaded polymersomes treated rats were significantly lower than those of other groups, showing the efficacy of co-loaded polymersomes in reducing OA rats joint swelling.

The body weights of the rats were also monitored alongside joint width to evaluate the systemic safety and therapeutic effects of co-loaded polymersomes. As shown in Figure 5C, the average body weight of normal rats increased by 1.46-fold over 4 weeks, reaching 403 ± 5.80 g. In contrast, untreated OA rats exhibited a 1.48-fold increase in body weight, from 263.6 ± 9.80 g to 390.4 ± 10.20 g, although their final body weight remained significantly lower than that of normal rats.

Among the treated groups, OA rats treated with co-loaded polymersomes demonstrated the most significant recovery in body weight, which approached that of normal rats by the end of treatment. However, rats treated with free cordycepin or free PBA showed relatively lower body weights compared to untreated OA rats. The superior therapeutic effect of co-loaded polymersomes could be attributed to the enhanced effects of the combined compounds and the sustained release profile of the polymersomes.

#### 3.4.3 Inflammatory cytokine profile

**FIGURE 6:**
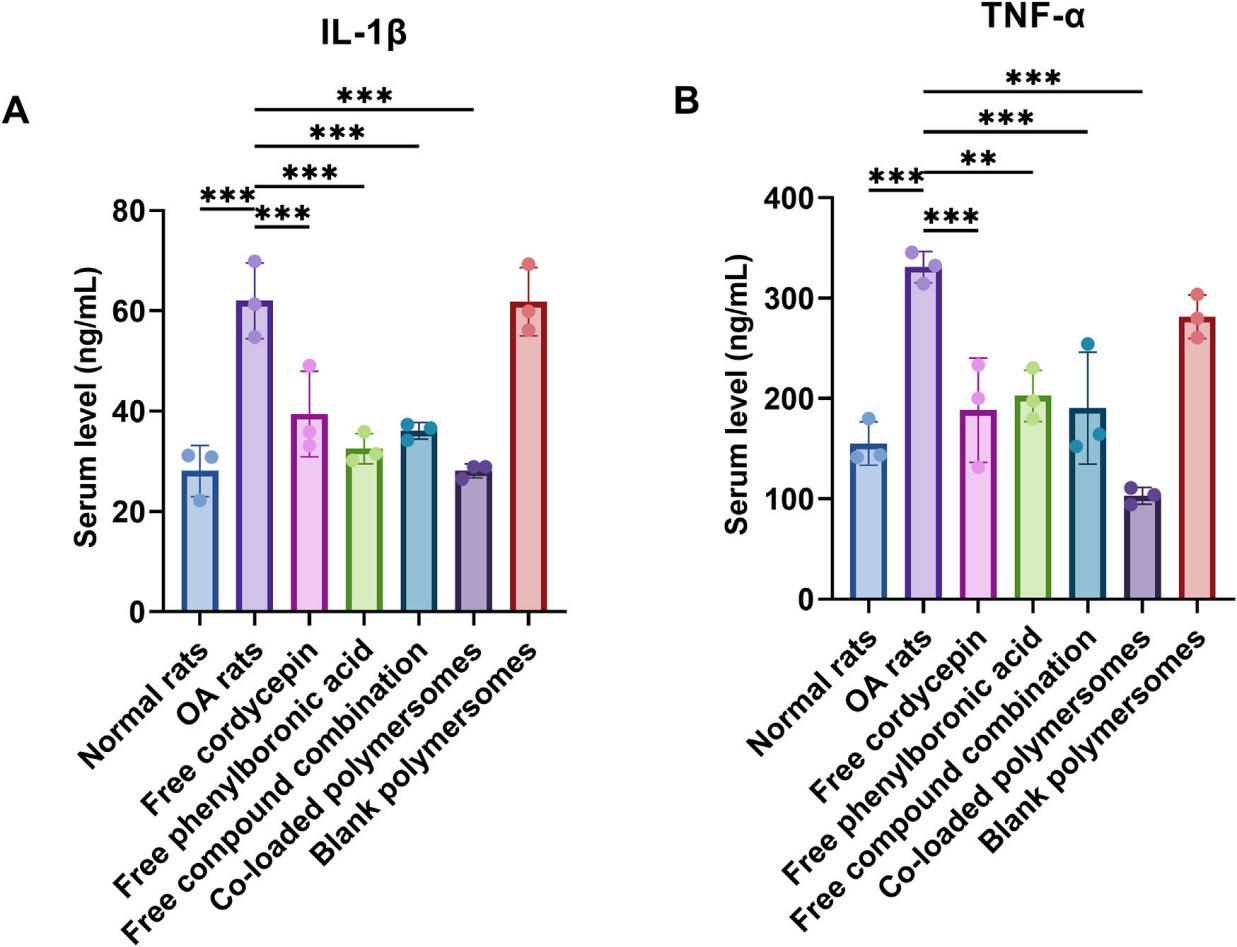
Therapeutic effects of different treatment groups on pro-inflammatory cytokine levels and cartilage histology in an OA rat model. **(A,B)** Serum levels of pro-inflammatory cytokines at day 28 after 4-week treatments with various samples. The levels of IL-1β **(A)** and TNF-α **(B)** were measured using ELISA kits. *p < 0.05, **p < 0.01, and ***p < 0.001 compared with OA model rats (n = 3).

To evaluate the anti-inflammatory effects of co-loaded polymersomes, serum levels of pro-inflammatory cytokines, including IL-1β and TNF-α, were detected using ELISA. As shown in Figures 6A,B, untreated OA rats had significantly elevated levels of IL-1β and TNF-α, contributing to abnormal cartilage matrix metabolism and increased cell apoptosis.

After 4 weeks of treatment, the levels of IL-1β and TNF-α were significantly reduced in all treated groups compared to untreated OA rats. Among them, the administration of co-loaded polymersomes demonstrated the lowest levels of both cytokines, which were similar to those in the normal rat group. It was noteworthy that the serum level of TNF-α in co-loaded polymersomes group was even lower than those in normal rat group, although no statistically significant difference was found between these two groups. These results indicated that co-loaded polymersomes effectively reduced systemic inflammation in OA rats.

#### 3.4.4 Histomorphometric analysis

The cartilage morphology of knee joints in OA rats was observed using hematoxylin and eosin (HE) staining to further evaluate the therapeutic effects of various treatments. As shown in Supplementary Figure S5, the surface of articular cartilage in the normal rat group was flat and smooth, with neatly arranged chondrocytes. In contrast, untreated OA rats exhibited severe cartilage damage, characterized by numerous defects, disorganized chondrocyte layers, and reduced cell density. The structural abnormalities confirmed the successful establishment of the OA model.

After 4 weeks of treatment, OA rats treated with co-loaded polymersome showed significant improvement in cartilage morphology. The cartilage surface was smooth, and the chondrocytes were arranged in an orderly manner, resembling the structure observed in normal rats (Supplementary Figure S5). In contrast, OA rats treated with blank polymersomes showed negligible effects on cartilage repair, with a large number of defects on the cartilage surface and disorganized chondrocyte layers. Uneven cell distribution and hemorrhage were also detected, similar to that in untreated OA rat model. The groups treated with free cordycepin or free PBA showed moderate improvements in cartilage surface smoothness and chondrocyte organization. However, irregular cell distribution and macrophage infiltration were still observed. Compared with either free compound alone, the free compound combination group demonstrated a smoother cartilage surface and more regular chondrocyte arrangement, but small focal hemorrhages were still present. Taken together, co-loaded polymersomes were the most effective treatment in reducing pathological cartilage damage and promoting cartilage regeneration in OA rats. The combination of cordycepin and PBA within the polymersome delivery system contributed to this superior therapeutic outcome, likely due to their enhanced effect and sustained release properties.

## 4 Discussion

Our study demonstrated the therapeutic potential of co-loaded polymersomes incorporating cordycepin and PBA for the treatment of OA. By leveraging the combined anti-inflammatory and antioxidant properties of cordycepin and PBA, delivered via an optimized polymersome formulation, our approach effectively addresses the multifactorial pathophysiology of OA, including chronic inflammation, oxidative stress, and cartilage degradation.

The co-delivery of cordycepin and PBA using polymersomes offered several advantages over the administration of free compounds. The sustained release profile of both agents prolonged therapeutic effects, reduced dosing frequency and minimized potential adverse effects. Moreover, the co-delivery system facilitated compound-compound interactions between cordycepin and PBA, enhancing anti-inflammatory and antioxidant activities. This mechanism likely contributed to the superior therapeutic outcomes observed in treated OA rats. While combination therapies for OA have been previously explored, they often face challenges related to the co-delivery of agents with differing solubility and stability. The polymersome system employed in this study effectively overcame these limitations. By providing a versatile platform for the co-delivery of both hydrophilic and hydrophobic compounds, this system demonstrated improved stability, targeted delivery capabilities, and compatibility, which were consistent with our results (Sharma et al., 2020; Sun et al., 2023).

Elevated levels of pro-inflammatory cytokines, including IL-1β and TNF-α, contribute to cartilage destruction and synovial inflammation in OA (Schuerwegh et al., 2003; Liu S. et al., 2022). In our study, treatment with co-loaded polymersomes led to a marked reduction in IL-1β and TNF-α levels, achieving levels similar to those observed in healthy rats. These results indicated the efficacy of co-loaded polymersomes in reducing OA-related inflammation and preventing further cartilage damage. Cordycepin has previously been applied in the treatment of OA due to its anti-inflammatory properties (Ashraf et al., 2019), specifically its ability to inhibit the NF-κB signaling pathway. It was prioritized during formulation development due to its poor system stability. Efficient encapsulation was necessary to preserve its bioactivity *in vivo*. However, monotherapy with cordycepin may not be sufficient for effective OA management due to the complex pathology involving both inflammatory and oxidative stress pathways. While cordycepin targets inflammatory signaling, PBA contributes by neutralizing ROS, another major contributor to cartilage breakdown and synovial damage. Previous study demonstrated that superoxide dismutase (SOD)-loaded polymersomes effectively reduced ROS levels and synovial inflammation, yet lacked a specific anti-inflammatory agent targeting cytokine production (Gui et al., 2022). In contrast, our co-loaded polymersomes provided dual-action benefits that directly reduced ROS and inhibited cytokine signaling, simultaneously addressing inflammation and oxidative stress. Thus, while cordycepin was the primary agent requiring encapsulation optimization, both compounds serve as co-therapeutics with complementary mechanisms, offering a more comprehensive approach to OA treatment.

While the current *in vitro* release study was designed to mimic the oxidative stress characteristic of OA joints using physiologically relevant concentrations of H_2_O_2_, it did not account for the presence of serum proteins, which are known to adsorb onto nanoparticle surfaces, alter colloidal stability, and potentially accelerate drug release. The protein-nanoparticle interaction may also influence both blood circulation and release kinetics *in vivo*. To address this, future work will include release experiments conducted in PBS containing 10% fetal bovine serum (FBS) at 37°C, under both static and shaking conditions. It can elucidate the effect of serum components on drug release and better simulate the dynamic extracellular environment encountered in systemic administration.

To overcome the limitations of conventional delivery methods for joint-specific treatment, we explored intraperitoneal (IP) injection as an alternative to intra-articular (IA) administration. IA injections, though effective for localized delivery, are associated with risks such as joint infection, technical challenges, and the need for repeated administration due to rapid clearance from the joint space. In contrast, IP administration of co-loaded polymersomes offered a less invasive alternative, enabling systemic circulation and subsequent accumulation in inflamed joints. This approach allowed sustained therapeutic benefits without the drawbacks of frequent IA injectiions.

The biodistribution analysis demonstrated that co-loaded polymersomes selectively accumulated in inflamed knee joints following IP administration. Fluorescence signals were detected in the joints for up to 24 h post-injection, indicating efficient targeting and prolonged retention at the site of inflammation. This accumulation was likely influenced by the increased vascular permeability characteristic of inflamed tissues (Au et al., 2021; Zhong et al., 2022; Liu et al., 2023), which is somewhat similar to the enhanced permeability and retention (EPR) effect observed in tumors. Although the EPR effect is less pronounced in OA compared to cancer, the deregulated angiogenesis and leaky blood vessels characteristic of inflamed joints may still facilitate the passive accumulation of nanoparticles, such as polymersomes, at the inflammation site. This mechanism demonstrated the potential to leverage inflammation-induced vascular changes to enhance the therapeutic targeting of nanoparticles in OA treatment. Efficient targeting of inflamed joints is important for maximizing therapeutic efficacy while minimizing systemic side effects. In this study, biodistribution analysis confirmed the selective accumulation of co-loaded polymersomes at OA sites following IP injection.

The co-loaded polymersomes effectively reduced joint swelling and restored body weight in OA rats during the 4-week treatment period, achieving levels similar to those of healthy control rats. This superior efficacy, compared to free compounds, was likely due to the combined effects of cordycepin and PBA, as well as the sustained release provided by the polymersomes. The recovery in body weight also indicated reduced systemic inflammation and enhanced overall therapeutic outcomes.

Histological analysis of cartilage tissue revealed that co-loaded polymersomes significantly promoted cartilage regeneration, resulting in a smooth cartilage surface with an organized chondrocyte arrangement comparable to that of healthy controls. In contrast, untreated OA rats and those receiving blank polymersomes exhibited significant cartilage damage, characterized by surface defects and disorganized chondrocyte layers. The observed cartilage regeneration in the co-loaded polymersome-treated group can be attributed to the sustained release of therapeutic agents, which provided continuous exposure at the site of OA.

Currently, growth factor-(Geiger et al., 2018; Au et al., 2021) or stem cell-based (Harrell et al., 2019; Chen et al., 2023) therapies demonstrate notable benefits for cartilage repair, but they often involve high costs and potential safety concerns. In contrast, the combination of cordycepin and PBA provides a less invasive, more cost-effective, and potentially safer alternative. Additionally, although hydrogels and microparticles have showed promise in promoting cartilage regeneration (Nguyen et al., 2021; Xu et al., 2022; Kalairaj et al., 2024), these approaches frequently encounter challenges related to scalability and consistent therapeutic delivery. In comparison, our results indicated that co-loaded polymersomes could offer an effective approach for enhancing cartilage regeneration.

Despite the promising results, some limitations existed. Although the co-loaded cordycepin/PBA polymersomes consistently outperformed the single-compound treatments in suppressing ROS production, reducing inflammatory cytokine levels, and alleviating OA-related weight loss, the current study did not include a formal pharmacodynamic interaction analysis. Accurate classification of drug combinations as synergistic, additive, or antagonistic requires a full concentration-response matrix analyzed using a validated model such as the Chou-Talalay combination index (CI) or isobologram analysis. Determining the synergistic interaction profile and combined ratio is particularly challenging in a complex *in vivo* animal model, given the distinct mechanisms of anti-inflammatory cordycepin and ROS-scavenging PBA. Moreover, the long-term efficacy and safety of co-loaded polymersomes was not assessed. These limitations will be addressed in future studies, which will include extended treatment schedules, combination-index profiling, and dose-escalation analyses to define the interaction landscape more precisely.

## 5 Conclusion

This study demonstrated the therapeutic potential of co-loaded polymersomes incorporating cordycepin and PBA for the effective treatment of OA. The co-loaded system exhibited significant anti-inflammatory, antioxidant, and cartilage-regenerative effects, providing a comprehensive approach to addressing the multifactorial pathophysiology of OA. The polymersome formulation offered a sustained release profile, enhanced joint targeting, and reduced adverse effects, resulting in superior therapeutic outcomes compared to free compounds alone or their combination. Overall, our results provided a promising foundation for the potential clinical translation of co-loaded polymersomes in OA treatment.

## Data Availability

The original contributions presented in the study are included in the article/Supplementary Material, further inquiries can be directed to the corresponding author.
