# Supplementary material for: Co-delivery of anti-inflammatory and antioxidant agents via polymersomes for osteoarthritis therapy
Source: Front Pharmacol. 2025 Jul 9;16:1635761. doi: 10.3389/fphar.2025.1635761 (PMC12283600; doi:10.3389/fphar.2025.1635761)
Supplement: Supplementary file 1 [file DataSheet1.docx]

Supplementary Material

Co-Delivery of Anti-inflammatory and Antioxidant Agents via Co-loaded Polymersomes for Osteoarthritis Therapy

Mengjie Rui^1^, Li Wang^1^, Ke Mi^1^, Yinfeng Li^1^, Naying Fang^1^, Yingying Ge^1^, Qiuqi Feng^1^, Yaqi Luo^1^, Chunlai Feng^1,2^*

^1^ School of Pharmacy, Jiangsu University, 301 Xuefu Road, Zhenjiang, 212013, Jiangsu Province, China

^2^NHC Key Laboratory of Diagnosis and Therapy of Gastrointestinal Tumor, Gansu Provincial Hospital, Lanzhou, 730000, Gansu Province, China

# HPLC Method development

The wavelength of maximum absorption of cordycepin and PBA after scanning was found to be 260 nm (Figure S1). Figure S3 depicts the chromatograms obtained for the analyzed standard solutions of two compounds using the developed method. As shown in Figure S2, cordycepin and PBA eluted well and showed separation from the blank polymersome materials. The retention time of 3.419 min for cordycepin and 5.255 min for PBA provided a faster determination.

**Figure** S1. UV-absorption spectra of cordycepin and PBA from 200 to 800 nm (C: cordycepin; B: phenylboric acid)

**Figure** S2. Chromatograms showing the specificity of HPLC method. (A) Two compounds; (B) the blank polymersomes.

# HPLC Method validation

## Linearity of HPLC method

The linearity of the calibration curves were determined by plotting the results from standard solutions at different concentrations (Figure S3). The standard curve and linear range were obtained as shown in Table S1. There was a good linear relationship over a wide concentration range.

**Figure** S3. Calibration curves of cordycepin (A) and PBA (B).

**Table** S1. Calibration curves for two compounds

| Peak number | Analyte | Calibration curve | R^2^ | Linear range (µg/mL) | LOD  (µg/mL) | LOQ  (µg/mL) |
| --- | --- | --- | --- | --- | --- | --- |
| 1 | cordycepin | *Y*=38.565*X* + 432.8 | 0.9991 | 12.5-125 | 0.063 | 0.191 |
| 2 | PBA | *Y* = 3.6113*X* - 12.176 | 0.9992 | 50-500 | 0.864 | 2.620 |

## Repeatability, stability, and precision of HPLC method

To validate the repeatability of the method, five different sample solutions from the same sample were injected into the HPLC system, and the RSD values were found to be 0.59% for cordycepin and 0.17% for PBA (Table S2).

The stability of the sample solutions was analysis at 0, 2, 4, 6, 8, 12, and 24 h. As shown in Table S3, two sample solutions were found to be stable for 24 h (RSD ≤ 1.17%).

The precision of the method was validated by their intraday variability. The intraday precision was evaluated by injecting the same standard solution six times. As shown in Table S4, the RSD values of precision were lower than 2%, indicating that the HPLC methods for two compounds are reliable and effective.

**Table** S2. Repeatability results of HPLC method

| Samples | Sample Number | | | | | | RSD (%) |
| --- | --- | --- | --- | --- | --- | --- | --- |
|  | 1 | 2 | 3 | 4 | 5 | 6 |  |
| Cordycepin (μg/mL) | 49.38 | 48.95 | 49.51 | 49.84 | 49.45 | 49.58 | 0.59 |
| PBA (μg/mL) | 203.41 | 203.40 | 204.01 | 203.72 | 204.27 | 203.52 | 0.17 |

**Table** S3. Stability results of HPLC method

| Samples | Time (h) | | | | | | | RSD (%) |
| --- | --- | --- | --- | --- | --- | --- | --- | --- |
|  | 0 | 2 | 4 | 6 | 8 | 12 | 24 |  |
| Cordycepin (μg/mL) | 48.95 | 50.51 | 49.84 | 50.45 | 50.1 | 49.64 | 50.58 | 1.17 |
| PBA (μg/mL) | 203.84 | 203.76 | 204.41 | 204.45 | 203.86 | 203.92 | 204.62 | 0.17 |

**Table** S4. Intraday precision results of HPLC method

| Samples | Sample Number | | | | | | RSD (%) |
| --- | --- | --- | --- | --- | --- | --- | --- |
|  | 1 | 2 | 3 | 4 | 5 | 6 |  |
| Cordycepin (μg/mL) | 48.75 | 49.68 | 48.58 | 49.84 | 50.21 | 50.63 | 1.16 |
| PBA (μg/mL) | 203.56 | 204.12 | 203.93 | 204.45 | 204.07 | 204.8 | 0.21 |

# ^1^H NMR spectra of polymer mPEG2k-PCL5k

The purified mPEG-PCL was analyzed using nuclear magnetic resonance (NMR) spectroscopy in deuterated chloroform. 1H NMR(400 MHz, CDCl3) δ, ppm: 1.36 (s, 2H), 1.64 (s, 4H), 2.31 (s, 2H), 3.38 (s, 3H), 3.64 (s, 4H), 4.04 (s, 2H).


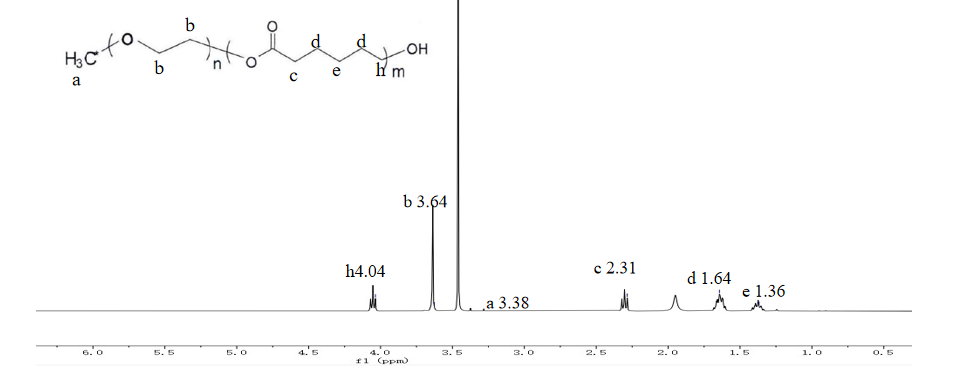


**Figure** S4. ^1^H NMR spectra of polymer mPEG2k-PCL5k.

# Histology
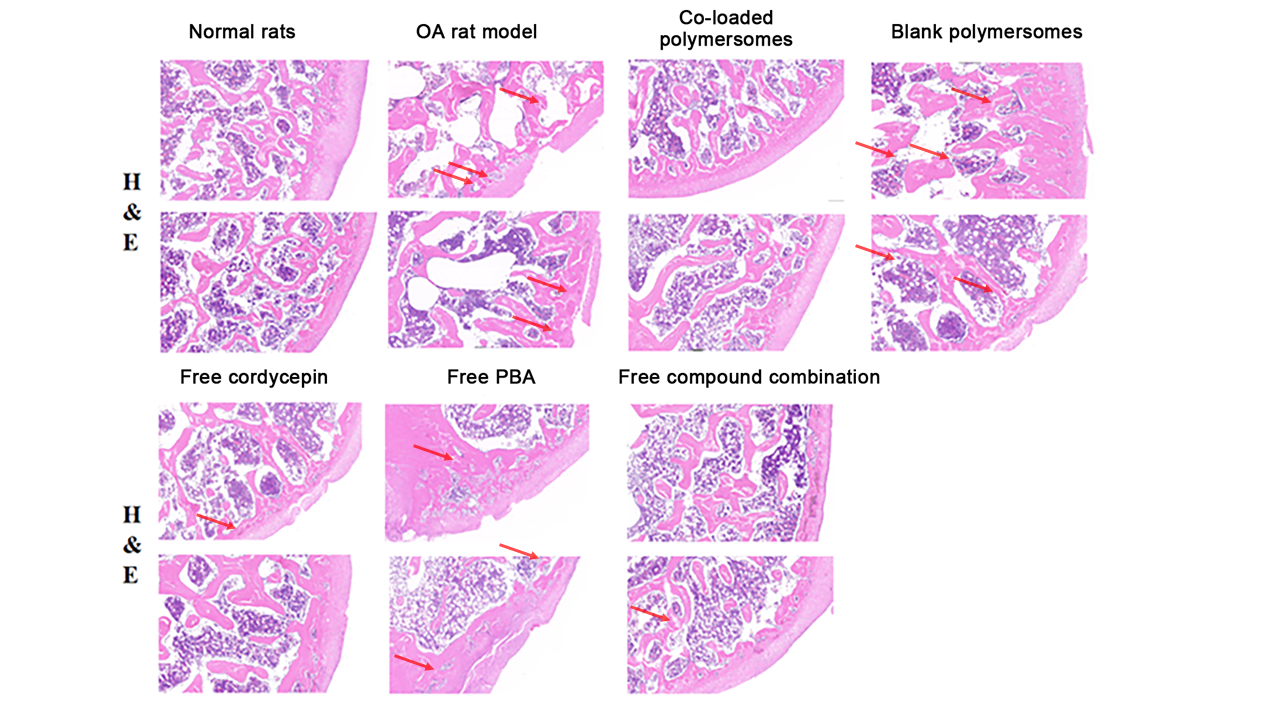


**Figure** S5. Histological images of cartilage from rat knee joints following 4-week treatments. Histological sections from normal rats, OA model rats, co-loaded polymersomes group, blank polymersomes group, free cordycepin group, free PBA group, and free compound combination group were stained with hematoxylin and eosin (HE, ×200). Arrows show focal hemorrhages.
